# Medical education reform in Tajikistan: comparison of the conventional one-year family medicine residency program and the new two-year residency program for postgraduate medical education

**DOI:** 10.1186/s12909-021-02749-x

**Published:** 2021-05-28

**Authors:** Leah F. Bohle, Edgar Valencia, Greta Ross, Davlyatova Dilbar Dzhabarovna, Shakhlo N. Yarbaeva, Zukhra A. Kasymova, Helen Prytherch

**Affiliations:** 1 Swiss Tropical and Public Health Institute (Swiss TPH), Basel, Switzerland; 2 University of Basel, Basel, Switzerland; 3 Pontificia Universidad Católica de Chile, Santiago de Chile, Chile; 4 Independent Health Consultant, England Canterbury, UK; 5 Medical Education Reform Project Tajikistan, Dushanbe, Tajikistan; 6 Department of Family Medicine, Institute of Postgraduate Education in Healthcare of the Republic of Tajikistan, Dushanbe, Tajikistan

**Keywords:** Medical education reform, Family medicine, Tajikistan, Specialty program, Residency, Evaluation, Validation

## Abstract

**Introduction:**

The last two decades have seen a shift in former Soviet countries from highly specialized to more family medicine-focused systems. Medical education has slowly adjusted to these reforms, although the region is still at risk to have a chronic shortage of family doctors. This paper presents the evaluation of a new post-graduate family medicine program in Tajikistan, focused on competency-based training. The findings are relevant for policy makers, international organizations and practitioners participating in similar medical education reform programs.

**Methods:**

We employed a quasi-experimental control group design and compared intervention residents, control group residents with traditional training, and 1st year residents with no training in two outcomes, clinical knowledge and competencies. We employed two objective measures, a written multiple-choice question test (MCQT) and an Objective Structured Clinical Examination (OSCE), respectively. We report reliability and validity of the measures along with ANOVA, planned contrasts and effect size estimates to examine differences across groups.

**Results:**

We found statistically significant differences in both clinical knowledge and competencies between intervention and control groups. We also detected a large intervention effect size. Participants in the intervention outperformed control group participants in the two measures. Our analysis suggests that intervention and control group participants are comparable in terms of initial knowledge and competencies, strengthening the argument that the intervention caused the improvement in the program outcomes.

**Discussion:**

Receiving tailored training and structured opportunities to practice knowledge and competencies in clinical settings have a positive effect on the education of family medicine doctors in Tajikistan. Our results support curriculum reform and investment in medical education in the form of longer and supervised on-the-job preparation designed to be more in line with international standards. We discuss suggestions for future studies and potential requirements to inform replicability in other countries.

**Conclusion:**

Family medicine is well recognized as central to health systems throughout the world, but high quality residency training lags behind in some countries. Our study showed that investing in family medicine residency programs and structured training is effective in increasing critical clinical competencies. We encourage promoting comprehensive post graduate family medicine doctor training so that the goals of a family medicine centered health system are attainable.

## Introduction

To achieve universal health coverage and contribute to healthy populations, well-performing health systems, offering highest quality of care, are critical. After its independence in 1991, Tajikistan transitioned from a highly specialized health care system to a system based on family medicine (FM) [1]. Attention was given to the strengthening of primary health care services and family medicine through a health systems approach.

The focus on medical education reform, to ensure a supply of well-trained family medicine (FM) doctors, has been among the main health reform priorities of the Ministry of Health and Social Protection of the Population of the Republic of Tajikistan’s (MoHSP) 2010–2020 Strategy [2, 3].

Tajikistan, with support of the European Union, the World Health Organization (WHO) and the Swiss Agency for Development and Cooperation (SDC) has invested into improving and scaling up FM in the country through various activities, albeit the country still faces a shortage of FM doctors [4, 5]. Until 2013, only a conventional way of training FM doctors had been established referred to as “Internatura”, which comprised an unstructured one-year residency, following graduation from medical school. The duration and content of this training is not in line with international standards as set out by WONCA Europe and its teaching organization, the European Academy of Teachers in General Practice/Family Medicine [6] but is being offered until today. In 2016, the Tajik government issued a comprehensive Strategic Plan for FM in 2016 which advocated for “…post-graduate specialty training with decentralized and prolonged curriculum (at least from 2 to 3 years), and a system of continuing professional development based on a credit system “ [7]. To contribute to the above, the SDCs Medical Education Reform Project (MEP) was launched in the year 2009 and ended in 2019 [8, 9].

In 2013, the MEP, in close collaboration with a variety of stakeholders (for details see [10]) developed a new residency program for FM doctors more in line with international standards and as an alternative to the Internatura program [8]. The Post-Graduate Specialty Training (PUST) in Family Medicine in Tajikistan aims to improve the clinical knowledge and competencies of FM doctors, including their leadership and communication skills, eventually leading to an increase in quality of care, contributing to a stronger health system, and better health of the Tajik population. The theoretical training is provided by FM trainers and takes place at the PGMI based in Dushanbe as well as at several clinical training bases (CTBs). The PUST has been implemented as a formal MoHSP pilot in MEP project districts since September 2014, enrolling a new two-year cohort every year since then. It was the intention to pilot the new system in the specialty of Family Medicine with a view to then expanding this to other specialties. With the phasing out of the MEP in 2019, the PUST program was evaluated between November and December 2018.

The evaluation focused on what could be objectively measured, and compared levels of clinical knowledge and competencies between newly graduated two-year PUST program participants (i.e. graduated PUST residents; referred to as “Ordinator-FDs” in previous publications) and the one-year conventional Internatura program (i.e. graduated Internatura residents; referred to as “Interns” in previous publications) for the specialization of FDs. Further, it assessed increment by comparing the graduated PUST residents with those who newly entered the PUST program (i.e. 1st year PUST residents; referred to as “1^st^ year Ordinator-FDs” in previous publications) [11].

Literature focusing on medical education reform of family medicine (or primary health care (PHC) reform) in Central Asia and neighboring countries is limited. Publications mostly exist in the form of government reports and decrees. Few documents outlining the reform in Kyrgyzstan [12, 13]; Uzbekistan [14]; and Russia [15, 16], or specific aspects of it, such as in Kazakhstan (e.g. the role of the Family Medicine Department in the reform) [17] could be identified. In addition very few publications, describing the importance of the training of residents in family medicine (and primary health care respectively) from Ukraine, Kyrgyzstan and Russia were found [18–20].

Peer-reviewed literature on aspects of medical education reform related to FM in former Soviet countries – including Eastern Europe and Central Asia - appears almost non-existent. Only very few peer-reviewed publications from Tajikistan are available, describing the medical education reform overall [3, 8]; the progress of the medical education reform activities as part of the MEP [8]; and the learning environment of PUST participants [21]. While two papers describe the nursing education reform and its effects in more detail [22, 23], only one peer-reviewed publication by Kempers et al. [10] is available so far, focusing specifically on the reform of the program for the specialization in FM. The economic investment case of the PUST program emphasizes its value to address the shortage of FM doctors expected in the future [10], including a brief presentation of the PUST evaluation results. So far, no detailed results of this evaluation were published, comparing the two different FM specialty programs in Tajikistan.

### Comparison of the residency programs in Tajikistan for the specialization in family medicine: the conventional Internatura and the newly reformed Post-University specialty training

The conventional one-year Internatura residency program, which is the traditional way of training since the Soviet era, attaches a graduate to a medical doctor at a primary healthcare centre. The FM doctor has not received formal training by a tutor and the graduate gains work experiences independently without a structured training schedule or established accountability system. No theoretical teaching is offered and the new graduates learn by themselves based on trial and error and by gradually acquiring work experience; residents can participate in conferences or seminars on their own expenses, which are however not part of the planned training program.

In comparison to the Internatura residency program, the PUST residency program in FM provides a structured approach and focuses strongly on training of clinical competence: It consists of 80% of practical work under clinical supervision by trained tutors, and 20% of lectures and teaching per week.

### Specifics of the Post-University specialty training

The PUST residency program in FM aims to provide a structured approach to the training of clinical knowledge and clinical competencies, with an emphasis on the latter. The program consists of 20 % of lectures and teaching per week and 80 % of practical work under clinical supervision.

For the practical clinical training, each resident is assigned to a tutor, who is an experienced FM doctor working at a rural health center or a CTB. The Post Graduate Medical Institute (PGMI) and MEP staff provide regular short refresher courses to tutors. Tutors and residents are visited on a monthly basis for monitoring by teachers from PGMI and RCFM. Graduates have their own list of patients and work under expert supervision. The PGMI delivers the training provided to all residents enrolled in the program. Further, the program includes capacity building of Faculty at the Tajik State Medical University on how to use Objective Structured Clinical Examination (OSCE)/In-Training Evaluation Report (ITER) to assess the clinical competency progress. In addition, to assure the continuous medical education (CME) and professional development of these newly educated FM doctors, an annual conference for students was introduced. Moreover, peer groups for newly graduated FM doctors were established, providing the opportunity for them to remain connected and keep to up to date – examining clinical issues encountered in practice and reviewing the evidence in a group discussion and reflection.

So far, the peer-reviewed literature focusing on the outcomes of medical education reform in former Soviet countries is very scarce. For Tajikistan only two peer-reviewed publications exist so far, focusing specifically on the reformation of the medical education of FM doctors in Tajikistan and as outlined above. This is the first paper comparing the conventional and the reformed new FM specialization programs as part of the medical education reform process in Tajikistan, assessing the differences in levels of clinical knowledge and competencies between residents. Results demonstrate the success of the medical education reform efforts related to the FM residency program. With international donors currently funding similar initiatives of medical education reform in the region (e.g. Kazakhstan, Kyrgyzstan, Uzbekistan; Ukraine; and Moldova), results of this evaluation are of interest to policy makers, donors and implementers in countries, with similar attention to family medicine and reform plans.

This article will 1. Compare the overall clinical knowledge and competencies level between participants in the conventional (Internatura) and reformed (PUST) family medicine specialization programs; and will 2. Identify specific clinical training domains in which the reformed FM PUST program may produce greater effect compared to the traditional FM specialization program.

## Methods

### Evaluation design

The evaluation employed a quasi-experimental design featuring three groups: 1) graduated PUST residents (*N* = 26), 2) graduated Internatura residents (*N* = 8) and 3) 1st year PUST residents (*N* = 20). All three groups are comprised of graduated medical students specializing or having newly specialized in family medicine. Participants in Group 1 received training under the PUST program; participants in Group 2 participated in the Internatura program, and participants in Group 3 were recently graduated medical students who newly entered the PUST program with no on-the-job training so far. Therefore, our target group (intervention) is group 1 while groups 2 and 3 serve as control groups. Each group was evaluated once, but due to the nature of the groups and the timing of the evaluation, the meaning of these evaluations differs across groups. While graduated PUST residents and graduated Internatura residents were evaluated after the specialization training (post-graduation, as in a *posttest*), 1st year PUST residents were evaluated before completing the training (as in a *pretest*).

This design allows the examination of differences between participants in the intervention (Group 1) and control groups (Groups 2 and 3). A positive difference between Group 1 versus Group 2 and between Group 1 versus Group 3 would both suggest intervention effect, given that other conditions for inferring causality are met.

In our study, random assignment to intervention condition was unfeasible due to logistic and budget constraints. However, comparison of the two control groups (pre-intervention and post-intervention) help cast light on initial differences that could confound the intervention effect with selection. No differences between these two untreated groups would support group comparability. We thoroughly address this and other design limitations for sustaining causal claims in the discussion section.

### Instrument development

The two program outcomes in the evaluation were clinical knowledge and competencies. The instruments comprised of a written multiple-choice question test (MCQT), focusing on the clinical knowledge of participants; followed by an Objective Structured Clinical Examination (OSCE) approach [24] focusing on clinical competencies (see Table 1). We maximized the validity of the two measures prior administration during instrument development and after administration by conducting psychometric analyses.

**Table 1:** OSCE stations

OSCE Stations	Thematic area	Topics covered
Station 1	Toddler with cough and fever	History of present illness Review of systems specific to an infant or toddler Communication; Counselling and challenge
Station 2	Headache	Focused physical examination Neurologic examination Communication
Station 3	Abdominal pain	Focused physical examination Communication
Station 4	Laboratory results interpretation	Main diagnosis History Next diagnostic steps
Station 5	Chest pain	Focused physical examination Communication and interpersonal skills Challenges

Instruments were developed based on nationally defined standards and competencies of a FM doctor [11] in combination with the highest disease burden in-country typically treated by a FM doctor [25] and complemented by expert opinion by consulting international and national medical experts, highly familiar with the Tajik context.

The multiple-choice questions were developed based on recommendations by the Yale Center for Teaching and Learning [26], the University of Waterloo Centre for Teaching Excellence [27] and Considine et al. [28]. Additional validated multiple-choice questions were chosen from a pool of questions from AMBOSS GmbH [29] and were adapted to the Tajik context. The final test consisted of 60 multiple choice questions each one with one correct answer and four distractors. Questions present applied problems based on the most common diseases treated by a FM doctor and the highest disease burden as well as the qualifications expected of FM doctors in Tajikistan [25].

For the OSCE, a total of five scenarios were developed with a focus on history taking and anamnesis aiming to assess attitudes and practices; three tracer diseases and lab results, aiming to assess examination, management and communication skills. Scenarios were drawn from internationally existing OSCES for FM doctors and adapted to the Tajik context. In addition, instructions and templates for examiners and simulators were developed.

MCQT and OSCE draft versions were shared with an international and national expert group for feedback; MCQT were reviewed by an expert panel for content relevancy and coverage [30] to provide content-based evidence of validity for the Tajik context. All instruments were translated into Tajik and translation was carefully checked by English-speaking Tajik medical staff in the MEP who carefully compared the English and Tajik versions for accuracy and ease of comprehension.

### Instrument validation

The purpose of the psychometric analysis prior to conducting further analysis was to examine the metric properties of items and scales in the MCQT and OSCE. Item properties include difficulty and discrimination, and scale properties are reliability and validity [31]. The analysis informed the exclusion of specific items to ensure higher reliability and validity. We followed standard practices from two psychometric frameworks: classical test theory (CTT) [32, 33] and exploratory factor analyses (EFA) [34, 35]. Using CTT, we discovered heterogeneous item difficulties ranging from perfectly easy items (0% incorrect answer) to items with 99% incorrect answer (too difficult). We discovered items with heterogeneous discrimination, with many items showing small (below 0.3) or even negative item-test Pearson correlation coefficient (discrimination index). We excluded items that were too easy or too difficult, and items with discrimination below 0.1.

Following, we conducted EFA to examine the internal structure of the two scales (MCQT, OSCE) and subscales (OSCE). In our analysis, we were interested in testing how the data complied with the intended unidimensional structure of the two measures. Thus, we tested for unidimensionality forcing a one factor solution. The exploratory factor analysis was conducted utilizing maximum likelihood estimation with no rotation. The maximum likelihood factor analysis produces a factor solution accounting for the common variance among items excluding random error and unique item variance. We discovered heterogeneous factor loadings with a few items per scale exhibiting low or negative factor loadings. Initially, we retained items with factor loadings higher than 0.3. We then decided to keep items with factor loadings higher than 0.2 avoiding sacrificing content coverage and undermining content representation and validity.

Table 2 presents the initial and final number of items per scale and subscale along with reliabilities before and after conducting CTT and EFA analyses. We only report MCQT total score because the test did not include enough items per trace-disease causing low reliability and poor factor structure concerns with subscales. We report OSCE total score and OSCE subscales because they contain enough number of items for adequate reliability and factor structure estimation. Overall, most scales and subscales work as intended. In specific, the results suggest two factors for the subscale “history of present illness” (OSCE station 1). However, only one of the factors shows acceptable reliability and correlates positively with other subscales. In addition, we excluded two OSCE subscales from further analysis due to low reliability and poor factor solutions: “counseling and challenge” (OSCE station 2) and “communication and interpersonal skills” (OSCE station 5). Thus, for all but three cases, the factor solutions comply with our assumption of unidimensionality confirming the correct functioning of the scales.

**Table 2:** Psychometric analysis results by scale

Scale	Name	Total Items	Initial Reliability	Retained Items	Final Reliability
MCQT	Overall clinical knowledge	60	0.4053	27	0.8523
OSCE	Overall clinical skill	138	0.9406	49	0.9005
OSCE 1	History of present illness (F2)^1^	12	0.6369	4	0.7312
OSCE 1	History of present illness (F2)^2^			7	0.6669
OSCE 1	Review of systems specific to an infant or toddler	20	0.8598	19	0.8713
OSCE 1	Communication	8	0.6535	6	0.7150
OSCE 2	Focused physical examination specific to headache	8	0.7052	6	0.7954
OSCE 2	Neurologic examination	11	0.8018	11	0.8018
OSCE 2	Communication	8	0.6972	6	0.7224
OSCE 2	Counseling and challenge^3^	3	0.3500	3	0.3500
OSCE 3	Focused physical examination	9	0.7496	8	0.7657
OSCE 3	Communication	9	0.6356	6	0.7750
OSCE 4	Main diagnosis	17	0.8257	15	0.8320
OSCE 4	History	8	0.7356	8	0.7356
OSCE 4	Next diagnostic steps	3	0.7137	3	0.7137
OSCE 5	Focused physical examination	12	0.7733	9	0.7969
OSCE 5	Communication and interpersonal skils^4,5^	9	0.4414	4	0.6120
OSCE	Communication	34	0.6923	18	0.7933

Note 1: “History of present illness” is the only criteria with a two-factor solution: one of the two solutions shows low reliability and correlates poorly with the other OSCE scales: it is not included in further analysis

Note 2: One item is a constant: everyone answered correctly, and it is therefore not included in the analysis

Note 3: Dropped subscale, poor initial and final reliability, and unsatisfactory factor solution

Note 4: One item is a constant: everyone answered correctly, and it is therefore not included in the analysis

Note 5: Dropped subscale, poor initial and final reliability, unsatisfactory factor solution

The reliability coefficient reported in our analysis is the Cronbach’s alpha internal consistency coefficient. All final scales and subscales reached acceptable reliabilities for research purposes (above 0.7 and closer to 1.0). In general, the psychometric analysis provided input for increasing the initial reliabilities. The most notable case pertains to the MCQT score with an initial Cronbach’s alpha coefficient of 0.40 and a final coefficient of 0.85.

The final step in our psychometric analyses involved estimating the Pearson correlation coefficient among scales and subscales. The scoring for scales and subscales is expressed in percentage of correct response. We expected positive correlations across scales and subscales, specifically, a positive and moderate correlation between clinical knowledge (MCQT) and clinical competencies (OSCE), and positive correlations among OSCE subscales. Overall, the data conformed with our expectations. The correlation between MCQT and OSCE was *r* = 0.5 (*p* < 0.001). The correlation coefficients among OSCE subscales ranged from − 0.08 (virtually zero, not statistically significant) and 0.76. These empirical results support the reliability and validity of the MCQT and OSCE scores for evaluation purposes.

### Recruitment strategy

All residents who newly entered the PUST program, and those who newly graduated in the PUST program or Internatura in 2018, were invited to participate. Recruitment of graduated Internatura residents proved challenging, with some who could not be reached, others who had left Tajikistan and some on maternity leave. Following the invitation, a total of 54 participants participated in the MCQT, and a total of 50 participants in the OSCE (Table 3). Four participants were no longer available during the assessment due to a variety of reasons, including maternity leave and government services. The recruitment strategy produced fewer participants identified as female (43%) than male (57%), and there were differences in the distribution of participants by biological sex across groups. The proportion of female 1st year PUST residents, graduated Internatura residents, and graduated PUST residents was 50, 62.5, and 31% respectively.

**Table 3:** Sample and sample size

Group	Description	Total Enrolment	MCQT Sample	OSCE Sample
1st year PUST residents	Graduated medical students newly entering the 2-year PUST program	*N* = 20	*N* = 20	*N* = 20
Graduated Internatura residents	Newly graduated FM doctors who underwent the 1-year unstructured work experience	*N* = 22	*N* = 8	*N* = 6
Graduated PUST residents	Newly graduated FM doctors who just completed the 2-year PUST program	*N* = 26	*N* = 26	*N* = 24

Note 1: Total enrolment refers to the number of residents who enrolled in the program

Participants were invited by email and followed-up by phone in case of no response. The invitation email included information on the purpose and outcome of the evaluation; and it was emphasized that participation is voluntary, results will be fully encrypted and may be published, and participation can be withdrawn any time and prior to the start of participation without any consequences. With participation consent was automatically provided. This was reiterated again prior to the start of the MCQT. Participants were compensated for travel costs but did not receive further incentives. The evaluation had been included in the project’s workplan agreed between SDC and MoHSP. Ethical approval was received from the MoHSP (Date: 1.11.2018; order number: 1–6/7747–7306).

### Evaluation procedure

The evaluation took place between November and December 2018 at PGMI facilities. The MCQT was administered by three invigilators, who received a short training and written instructions to read out to participants to ensure a standardized process. Inclusion criteria of invigilators included no previous or current affiliation to any of the FM programs. Participants were allocated a number and randomly assigned to different rooms and seated separately. The overall written exam took 2.5 h.

A total of four patient simulators were trained, based on individual scripts. OSCEs took place over 2 days and a total of 10 examiners were trained. Examiners came from medical institutions training Internatura and PUST residents. To reduce the Hawthorne effect, one examiner from each training institution were placed in one room, overseeing one scenario each. The grading was based on a template with a variety of pre-defined grading criteria; examiners were asked to compare grading results after each performance and come to a joint conclusion.

## Data analysis

We conducted a series of ANOVA to gather evidence about differences in the program outcomes across groups. The F statistic from ANOVA tests the null hypothesis of no difference. We report the F statistic and exact *p*-value. *P*-values indicate if a statistical model (i.e., the null hypothesis) fits the data. Following expert recommendation on the use of statistical significance [36], we report effect sizes using the eta-square coefficient. Eta-square indicates the proportion of variance explained by the group membership. Effect size informs about the practical significance of an effect regardless of the statistical significance [37]. While a trivial effect may turn into a statistically significant effect because a spurious large sample size, a non-statistically significant effect could hide a large important effect of an underpowered study. The larger the index, the more important is the difference. A prevalent guideline [38] proposes interpreting an eta-square above 0.01 as small, above 0.06 as medium, and above .14 as large effect. We expect a medium to large effect size in both clinical knowledge and competencies.

ANOVA is a first step to explore group differences because the F statistic is not informative of where these differences are (if any). We conducted planned contrasts to examine differences between specific pairs of groups. We compare graduated PUST residents vs. graduated Internatura residents and graduated PUST residents vs. 1st year PUST residents to determine intervention effect. Additionally, we compared graduated Internatura residents with 1st year PUST residents to ascertain about pre-intervention group comparability. We present the difference between groups in the same metric of the original variables (percentage of correct answers) along with statistical significance.

## Results

Table 4 shows the results from conducting ANOVA on the two program outcomes by group. The table also shows the size effect coefficient (eta-square) and the coefficients from the planned contrasts.

**Table 4:** Differences in clinical knowledge and clinical competencies across groups

Scale	Outcome	F	df	*p*-value	eta^2^	Graduated PUST residents vs. graduated Internatura residents	Graduated PUST residents vs. 1st year PUST residents	Graduated Internatura residents vs. 1st year PUST residents
MCQT	Clinical Knowledge	7,17	2,53	.0018	.219	.217*	.177*	−0,04
OSCE	Clinical Competencies	9,8	2,51	.0003	.294	.251*	.198***	−.053

*p < 0.05; **p < 0.01; ***p < 0.001

Results from ANOVA suggest that differences in clinical knowledge (MCQT) by group were statistically significant (F(2,53) = 7.17; *p* = .0018). The effect size of the difference by accounts of the eta-square coefficient was rather large (eta-square = .22). The planned contrast shows that graduated PUST residents outperformed both graduated Internatura residents (F(1,51) = 8.45; *p* = .0054) and 1st year PUST residents (F(1,51) = 10.410; *p* = .002). Also, there was no statistically significant difference between graduated Internatura residents and 1st year PUST residents in their clinical knowledge (F(1,51) = .270; *p* = .608). Similarly, results from ANOVA suggest that differences in clinical competencies (OSCE) by group were statistically significant (F(2,49) = 9.80; *p* = 0.0003). The size of the effect was large (eta-square = .30). Planned contrast shows that graduated PUST residents outperformed both graduated Internatura residents (F(1,47) = 10.49; *p* = 0.0022) and 1st year PUST residents (F(1,47) = 14.85; *p* = .0004). There was no statistically significant difference in clinical competencies between graduated Internatura residents and 1st year PUST residents (F(1,47) = .45; *p* = .50).

Table 5 portrays the results from ANOVA on OSCE criteria by group. We observed the same general pattern than above with statistically significant differences in six criteria: history of present illness (station 1), review of systems specific to an infant or toddler (station 2), neurologic examination (station 3), focused physical examination (station 3 and station 5), and main diagnosis (station 4). The sizes of the effects are large because the eta-square is above .16 in every of these six criteria. There was no evidence of differences in communication (station 1, 2, 3 and overall), focused physical examination (station 2), history (station 4), and next diagnostic steps (station 4). Notably, there was only one statistically significant difference between graduated Internatura residents and 1st year PUST residents: main diagnosis (station 4).

**Table 5:** Differences in OSCE criteria across groups

OSCE Station	Criteria	F (2,51)	***p***-value	eta^**2**^	Graduated PUST residents vs. graduated Internatura residents	Graduated PUST residents vs. 1st year PUST residents	Graduated Internatura residents vs. 1st year PUST residents
OSCE 1	History of present illness	5.44	.0075	.190	.220*	.201**	−.019
OSCE 1	Review of systems specific to an infant or toddler	10.66	.0002	.312	.224*	.262**	.037
OSCE 1	Communication	1.10	.3421	.045	−.0833	.105	.188
OSCE 2	Focused physical examination specific to headache	1.30	.2810	.052	−.229	−.093	.136
OSCE 2	Neurologic examination	10.74	.0001	.313	.215*	.276**	.060
OSCE 2	Communication	1.41	.2533	.057	.076	.120	.044
OSCE 3	Focused physical examination	6.34	.0036	.212	.333**	.210**	−.122
OSCE 3	Communication	1.61	.2098	.064	.201	.137	−.063
OSCE 4	Main diagnosis	5.22	.0090	.181	.322**	.062	−.260*
OSCE 4	History	1.41	.2541	.056	.177	.089	−.087
OSCE 4	Next diagnostic steps	1.34	.2710	.054	.236	.119	−.116
OSCE 5	Focused physical examination	5.12	.0098	.178	.310**	.184*	−.125
OSCE	Communication	2.36	.1052	.091	.099	.129	.029

*p < 0.05; **p < 0.01

## Discussion

Current graduates pursuing the family medicine specialty in Tajikistan undergo a conventional training comprised fundamentally of 1 year of on-site work experience (i.e. Internatura program). Our study evaluated a program for training in the specialty of family medicine that better adheres to international guidelines (i.e. PUST program) and comprises lectures and on-site clinical work under supervision. The two programs greatly differ in length and intensity of the training the future family medicine doctors receive. The two-year PUST residency program provides a structured approach and has put great attention on improving clinical competencies (i.e., with a ratio of clinical competencies to theoretical training of 80%:20%), under the guidance of a trained FM tutor. Aspects of clinical competencies training include all steps of patient care such as history taking, examination, diagnostic steps, interpretation of results, diagnosis and treatment, and follow-up.

The results of the evaluation support the assumption that the PUST program contributes to an increase in clinical knowledge and competencies. Receiving theoretical training by skilled tutors, and being able to implement the knowledge in clinical practice, seems to be a successful approach. Likewise, results from the OSCE show that graduated PUST residents outperform graduated Internatura residents, as well as participants who newly entered the PUST program in overall clinical competencies. The PUST graduates perform significantly better in six criteria. As a whole, the results support that the PUST program is effective by a large margin than the current FM specialization training. In fact, the evaluation results showed that compared to the PUST training, the current Internatura program does not enable doctors to improve their clinical competence or raise their theoretical knowledge over and above what they learned at university.

There were a few aspects in which we report unexpected results, particularly pertaining clinical competencies. The significant difference in providing the main diagnosis after interpretation of laboratory results (station 4) between graduated Internatura residents and 1st year PUST residents may be explained by the fact that newly graduated medical students have not been exposed to the disease indicated by the laboratory results to the same extent than have graduated Internatura residents. While general consultation and communication skills were included in the PUST training program, no formal training addressing specific communication with patients was included; it is therefore not surprising that no differences could be seen between the three groups. This finding demonstrates the need for PUST to introduce more practical training in communication scenarios.

The credibility of our conclusions rests on several evaluation design features, and we believe that the methodology used in this evaluation may also be of wider interest for other programs. The main strength of the study is the utilization of two objective measures. Both the MCQT and OSCE were developed in-house following rigorous standards. The reliability and validity of the two measures comply with widely accepted quality criteria that it is fully accounted in the study.

Another strength of this evaluation is the utilization of two control groups. An initial limitation of the study relates to the use of a quasi-experimental design and the expectation of inferring about program effect. The study compares three unmodified groups in a setting where random assignment of medical students to the different programs or stages within a program (1st year PUST residents; graduated PUST residents; and graduated Internatura residents) is unfeasible. In theory, lack of random assignment prevents effectively ruling out initial differences across groups, increasing the chance of a confound between program effect and selection [39, 40]. However, to our knowledge, the initial characteristics of the medical graduates in the study do not differ between Internatura and PUST program participants. First, the medical training prior to enrolment in the Internatura/PUST program remained the same. Medical students’ pre-training knowledge and competencies seem equivalent across three study groups. Second, we are not aware of changes in the setting or context that account for differences in the enrolment of participants to Internatura or PUST program. Although stipends and medical books were provided to PUST residents, which may attract participants, the Internatura program is shorter and allows graduates to earn a salary earlier on. Overall, it is accepted that family medicine does not particularly attract better students, as these choose specialty programs with higher prestige. In fact, the PUST program is neither less nor more attractive for medical students than the Internatura program. Third, as expected, the comparison between graduated Internatura residents and 1st year PUST residents (the control groups having received no supervised structured training), show similar levels of clinical knowledge and competencies. For these reasons we believe selection is not a threat for interpreting these findings as program effect. Lastly, the study also features strategies to enhance statistical power. Our total number of participants seem adequate for detecting program effect, but the design is unbalanced. One group (i.e. graduated Internatura residents) just reaches the minimum number of participants for conducting ANOVA. Although ANOVA is relatively strong against unbalanced designs, an underpowered design is a consideration when trying to detect a small effect. We conducted planned contrasts as a way to increase statistical power. Our contrasts are explicitly aligned with our evaluation design and goals and helps minimize *phishing* and *type I error* [39]. We also increased statistical power by means of improved reliability of our measures. Our psychometric analysis greatly improves the reliability of the MCQT and OSCE subscales. Despite the unbalanced design, we detected consistently positive medium to large size effects across MCQT, OSCE and a number of the OSCE subscales providing greater support to our conclusions about a positive and relevant PUST program effect.

We cannot guarantee the generalizability of our findings, as participation was voluntary, and not all group members attended to the full evaluation. We were not able to examine whether participants with MCQT or OSCE data were different than those with no data. Anecdotal evaluation evidence suggests that participants in the evaluation might not differ from those that were not part of the evaluation and that non-participation was due to chance (for instance, maternity leave, migration). A future evaluation should cast light on the extent to which these findings also apply to future cohorts of PUST residents and different contexts.

### Future studies

Detecting the effect of a medical educational reform is challenging because changes take time to have an observable effect on critical outcomes. Our findings are promissory because the size of the effect in knowledge and competencies is substantial. A first interesting follow-up issue pertains to the extent to which differences between Internatura graduates and PUST graduates include other outcomes besides clinical knowledge and competencies. Future studies could monitor on-the-job performance between these two types of FM doctors. A second relevant aspect not considered in our study pertain to impact on patients’ variables, for instance, quality of care, health-related quality of life or DALYs. A third issue is the effect on job conditions. For instance, does the better training impact on the employability and career progress of the graduated from FM specialty? Do PUST graduates show less attrition and mobility than Internatura graduates and does recognition of the FM specialty change as a result of a high quality training? An additional question relates to the broader goal of the FM reform: Does the improved training of FM doctors help attract more candidates to the FM specialty and reduce the shortage? Fourth, more research is required to understand how our specific implementation of the PUST program in the Tajik context is achieving better outcomes than the traditional Internatura. Despite the many positive findings, how can the PUST program be improved? Furthermore, is the effect homogeneous across participants, for instance, by gender or another policy-relevant variable?

With the reformation of the family medicine specialty program we intended to showcase that longer specialty training closer to international standards is essential for quality education of doctors - even if 2 years is still considered too short in international comparison. However, there was an undercurrent of resistance to expanding the length of all specialty trainings due to concerns about cost implications. Our findings, and coherent with recent publications [8, 10], underscore the importance for more and strong evidence to show the benefits of longer, and higher quality training, brings savings to the health system in the long run. To replicate the effects of a reformed residency program (e.g. the PUST) in different settings or countries, requires transparent sharing of challenges and best practices. It is suggested to accompany similar programs with an integral process and impact evaluation [41] to be able to better inform advocacy for political buy-in, replication and sustainable funding at all levels.

## Conclusion

To develop and maintain a strong and sustainable primary health care system requires that a substantial part of graduating doctors go into primary care, and are supported with appropriate and high quality training to work as competent family doctors. The training of family medicine doctors is critical to ensure the success of health reforms offering the highest quality of care, the promotion of healthy populations, and in achieving universal health coverage.

The demonstrated cost-effectiveness [10] and overall outcomes suggest moving from conventional residency programs, such as the Internatura, towards more structured programs in line with international standards, such as the PUST program in Tajikistan. The success of the changes demonstrated in our study highlights the demand for proper modification of the medical education curriculum, clear description of roles and responsibilities, the training of staff, and the allocation of educational resources including on-site facilities for clinical practice, as well as buy-in by all relevant stakeholders. The reformation of the residency program in family medicine is a successful example, which can also be expanded to other specialties, and which is of interest to governments, international collaboration agencies, and practitioners in countries trying to conduct similar health reforms.

## Data Availability

The datasets used and/or analysed during the current study are available from the corresponding author on reasonable request.

## Abbreviations

ANOVA: Analysis of Variance
CME: Continuous Medical Education
CTB: Clinical Training Base
CTT: Classical Test Theory
EFA: Exploratory Factor Analyses
FM: Family Medicine
ITER: In-Training Evaluation Report
MCQT: Multiple-Choice Questions Test
MEP: Medical Education Reform Project
MoHSP: Ministry of Health and Social Protection of the Population of the Republic of Tajikistan
OSCE: Objective Structured Clinical Examination
PGMI: Post Graduate Medical Institute
PHC: Primary Health Care
PUST: Post-Graduate Specialty Training
RCFM: Republican Clinical and Family Medicine Training Centre
SDC: Swiss Agency for Development and Cooperation
WHO: World Health Organization
